# The Effects of Synbiotics on Dextran-Sodium-Sulfate-Induced Acute Colitis: The Impact of Chitosan Oligosaccharides on Endogenous/Exogenous *Lactiplantibacillus plantarum*

**DOI:** 10.3390/foods12112251

**Published:** 2023-06-02

**Authors:** Yunjiao Zhao, Liangyu Xue, Shunqin Li, Tao Wu, Rui Liu, Wenjie Sui, Min Zhang

**Affiliations:** 1 State Key Laboratory of Food Nutrition and Safety, Tianjin University of Science & Technology, Tianjin 300457, China; zhaoyj9308@163.com (Y.Z.); shirley_xly523@163.com (L.X.); sqli18306891445@163.com (S.L.); lr@tust.edu.cn (R.L.); wjsui@tust.edu.cn (W.S.); 2 China-Russia Agricultural Processing Joint Laboratory, Tianjin Agricultural University, Tianjin 300384, China

**Keywords:** *Lactiplantibacillus plantarum*, chitosan oligosaccharides, colitis, short-chain fatty acids, cytokines, gut microbiota

## Abstract

In this work, *Lactiplantibacillus plantarum* (*L. plantarum*) isolated from mice feces (LP-M) and pickles (LP-P) were chosen as the endogenous and exogenous *L. plantarum*, respectively, which were separately combined with chitosan oligosaccharides (COS) to be synbiotics. The anti-inflammatory activity of LP-M, LP-P, COS, and the synbiotics was explored using dextran-sodium-sulfate (DSS)-induced acute colitis mice, as well as by comparing the synergistic effects of COS with LP-M or LP-P. The results revealed that *L. plantarum*, COS, and the synbiotics alleviated the symptoms of mice colitis and inhibited the changes in short-chain fatty acids (SCFAs), tumor necrosis factor-α (TNF-α), interleukin (IL)-1β, IL-6, IL-10, and myeloperoxidase (MPO) caused by DSS. In addition, the intervention of *L. plantarum*, COS, and the synbiotics increased the relative abundance of beneficial bacteria *Muribaculaceae* and *Lactobacillus* and suppressed the pathogenic bacteria *Turicibacter* and *Escherichia-Shigella*. There was no statistically difference between LP-M and the endogenous synbiotics on intestinal immunity and metabolism. However, the exogenous synbiotics improved SCFAs, inhibited the changes in cytokines and MPO activity, and restored the gut microbiota more effectively than exogenous *L. plantarum* LP-P. This indicated that the anti-inflammatory activity of exogenous LP-P can be increased by combining it with COS as a synbiotic.

## 1. Introduction

Inflammatory bowel disease (IBD) is an aberrant immune-mediated gut inflammation that is mainly triggered by genetic, environmental, infectious, and immune factors [1]. IBD includes ulcerative colitis (UC) and Crohn’s disease (CD). UC is a chronic inflammation of the submucosa and epithelium in the colon. The recommended medication for treating UC is aminosalicylic acid, such as mesalazine, which is usually substituted by corticosteroids and immunosuppressants when it shows ineffectual treatment in patient. However, these medications have drawbacks such as low clinical effectiveness, immune resistance, and ambiguous long-term safety [2]. Due to the crucial role that the gut microbiota plays in the onset and development of UC, probiotics, prebiotics, and synbiotics, which primarily affect the gut microbiota, have received a lot of attention as alternative therapy options [3].

Probiotics are microorganisms that are beneficial to host health when consumed in sufficient amounts [4], which play roles in human health and have preventive, palliative, and therapeutic effects on diseases such as lactose intolerance, diabetes and obesity, acute diarrheal disease, inflammatory bowel diseases and irritable bowel syndrome, cancer, cardio-vascular diseases, urogenital infections, allergy [5], and host immunity [6]. Probiotic fermented foods also hold superior antioxidant activity and antimutagenicity [7]. Prebiotics are short-chain carbohydrates that cannot be broken down in the gastrointestinal tract but can be utilized by the host’s beneficial gut microbiota to produce a range of healthful active substances [8]. Synbiotics are specialized mixtures of probiotics and prebiotics that collaborate to improve host health by affecting the gut microbiota [9,10]. *Lactobacillus*, as one of the most common probiotics utilized to form synbiotics, has a wide range of physiological effects and health advantages that improve the mucosa, immune system, and gut microbiota [11]. Indigestible oligosaccharides have been recognized as the primary prebiotics [12] that can improve the metabolism, immunity, and intestinal health protection of host individuals, resulting from the capacity to encourage the growth of beneficial gut microbiota [13].

It has been demonstrated that synbiotics combined with *Lactobacillus* and oligosaccharide can alleviate colitis. For example, Liao et al. [14] treated colitis mice with synbiotics composed of fructo-oligosaccharides and probiotics containing *Lactobacillus* and found that synbiotics relieved symptoms of colitis by correcting gut microbial imbalance and by enhancing the intestinal barrier and intestinal immunity. A synbiotic constituted with *Lentinus edodes* dietary fiber and *L. plantarum* was demonstrated to regulate the Th17/Treg balance and oxidative stress, to stimulate the butyric acid production in feces, to maintain intestinal homeostasis, and to repair intestinal epithelial damage in colitis mice [15]. Raffaele et al. [16] illustrated that the prevention and therapy of mice colitis were significantly promoted by the use of synbiotics composed of *L. paracasei*, fructo-oligosaccharide, and arabinogalactan, which reinforced intestinal defense and restored the intestinal barrier in mice. However, there is a lack of research on the promoting effect of synbiotics composed of the same oligosaccharide on the same species of *Lactobacillus* isolated from different origins.

In this work, we selected two strains of *L. plantarum* as probiotics, including endogenous *L. plantarum* isolated from mice feces and exogenous *L. plantarum* isolated from pickles. Meanwhile, chitosan oligosaccharides (COS) were chosen as prebiotic to combine with endogenous and exogenous *L. plantarum* to obtain synbiotics. *L. plantarum*, COS, and the synbiotics were used as an intervention in dextran-sodium-sulfate (DSS)-induced acute colitis mice to investigate their effects on restoring gut metabolism, immunity, and microbiota, as well as the contrasting impact of COS on endogenous and exogenous *L. plantarum*. The purpose of this study is to compare the differences in anti-inflammatory activity between synbiotics consisting of endogenous or exogenous *L. plantarum* and the same prebiotic COS, as well as to compare the synergistic effects of COS with endogenous or exogenous *L. plantarum*. The study also aimed to provide guidance for the production and use of synbiotics, as well as the efficient alternatives to treat UC.

## 2. Materials and Methods

### 2.1. Strains and Chitosan Oligosaccharides

Endogenous *L. plantarum* was isolated from C57BL/6 mice feces. Briefly, fresh feces were collected in a sterile environment, weighed at 1 g, and then fully homogenized in 99 mL of sterile physiological saline. In total, 100 μL of homogeneous liquid that has been appropriately diluted was plated onto the De Man Rogosa Sharpe (MRS, Qingdao Hi-Tech Industrial Park Haibo Biotechnology Co., Ltd., Qingdao, China) plates, which was subsequently put in an incubator at 37 °C for 72 h. Strains with colony morphology of typical lactic acid bacteria colonies were selected to be performed by a three-zone scribe on an MRS plate three times to be purified. Gram-positive and catalase-negative isolates were accepted as the probable lactic acid bacterial [17], and they were identified via molecular identification. Briefly, the DNA of isolated strains were extracted using a bacterial genomic DNA extraction kit (Beijing Solarbio Science & Technology Co., Ltd., Beijing, China) according to the manufacturer’s instruction and amplified via polymerase chain reaction (PCR) using uni-versal primers 27F and 1592R. PCR amplifications were carried out at T100 Thermal Cycler (Bio-Rad, Hercules, CA, USA) with 25 μL of a PCR reaction mixture (2.5 μL of 10× PCR buffer, 0.5 μL of dNTP, 1 μL of primers, 0.5 μL of EX-Taq enzyme, and 1.5 μL of template DNA, fixed by ultrapure water), following these steps: pre-denaturation for 2 min at 94 °C, followed by 30 cycles of 94 °C for 45 s, 55 °C for 30 s, and 72 °C for 10 min [18]. PCR products were sequenced by Suzhou GENEWIZ Biotechnology Co., Ltd. (Suzhou, China). The results of sequence were compared using the Basic Local Alignment Search Tool (BLAST) software (http://blast.ncbi.nlm.nih.gov, accessed on 30 August 2021) algorithm at the National Center for Bio-technology Information (NCBI). A strain of *L. plantarum* was obtained, and it was chosen as the endogenous *L. plantarum*. Exogenous *L. plantarum* was isolated from pickles and provided kindly by Wecare Probiotics (Suzhou) Co., Ltd. (Suzhou, China). Endogenous and exogenous *L. plantarum* were labeled LP-M and LP-P, respectively. COS was generously provided by Dalian GlycoBio Company, Ltd. (Dalian, China), and its polymerization ranged from 2 to 6, with purity greater than 90%.

### 2.2. Strain Cultures and Synbiotic Preparation

LP-M and LP-P were cultured in MRS medium at 37 °C for 18 h to reach the end of the logarithm, harvested via centrifugation at 8000 r/min, resuspended in sterile saline, and centrifuged once more, and this process was repeated three times to remove the media, referring to the method of Yang et al. [19] and modified slightly. The suspension concentration of *L. plantarum* was adjusted to 5 × 10^11^ CFU/mL with sterile saline for further use. The COS was dissolved to a concentration of 30 mg/mL. Meanwhile, COS was weighed a certain quantity into the *L. plantarum* suspension to achieve a concentration of 30 mg/mL to obtain synbiotics [20].

### 2.3. Animal Experimental Design and Tissue Collection

**Figure 1:**
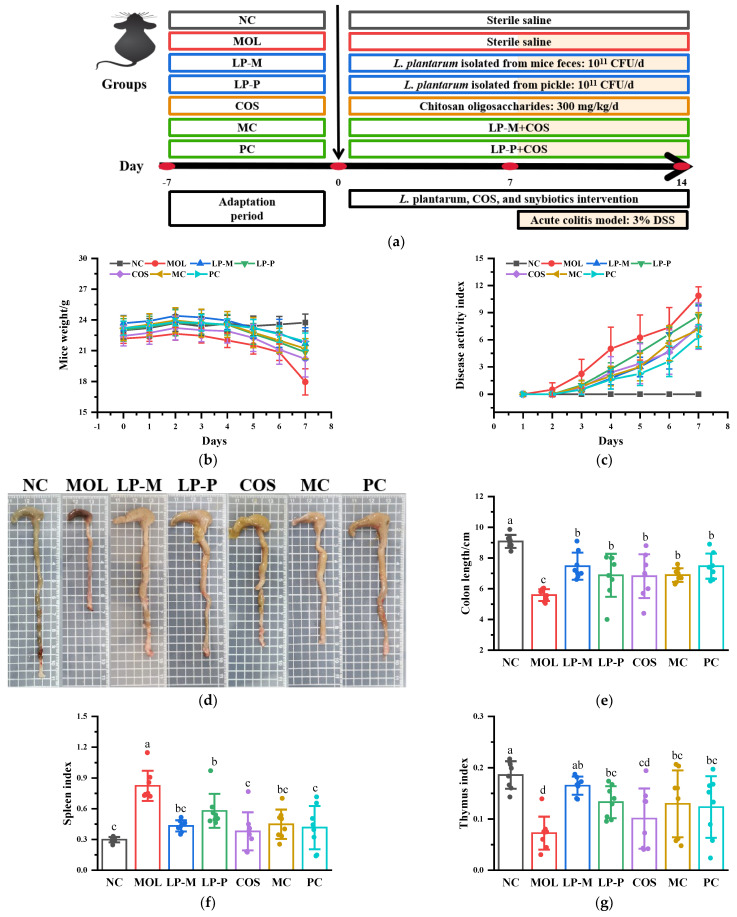
*L. plantarum*, COS, and synbiotics alleviated the DSS−induced colitis symptoms (n = 8). (**a**) The experimental protocol. (**b**) The changes in mice weight during DSS administration. (**c**) The change of disease activity index (DAI) during DSS administration. (**d**) A representative macroscopic photograph of the colon tissue. (**e**) The colon length. (**f**) The spleen index. (**g**) The thymus index. Statistical analysis was performed by one−way ANOVA with Duncan Test. The different letters above the columns indicated a significant difference (*p* < 0.05).

The animal experimental procedures were approved by the National Laboratory Animal Ethics Committee of China and in agreement with the Institutional Animal Care and Use Committee of the Tianjin University of Science and Technology (TUST 20221114). Fifty-six male C57BL/6J mice (six-week-olds, 20 ± 2 g), purchased from SPF (Beijing) Biotechnology Co., Ltd. (Beijing, China), were allowed to eat and drink ad libitum in a specific condition, which was free from pathogens, and with a temperature of 25 ± 2 °C, a humidity of 55% ± 5%, and 12 h light-to-dark cycles. After a week of acclimatization, the mice were divided into 7 groups of 8 mice at random. The mice in the normal control (NC) and model control (MOL) groups were gavaged with 0.2 mL of sterile saline once a day for two weeks, and five experimental groups were gavaged daily with 0.2 mL of *L. plantarum*, COS, and the synbiotics, respectively, for two weeks. According to the administration samples, the five experimental groups were named LP-M, LP-P, COS, MC (synbiotic combined of LP-M and COS), and PC (synbiotic combined of LP-P and COS). Dextran-sodium-sulfate (DSS, Dalian Meilunbio Co., Ltd., Dalian, China, molecular weight ranged from 36,000 Da to 50,000 Da) was substituted for drinking water for the mice for 7 consecutive days to induce the acute colitis (shown in Figure 1a). The mice’ body weights were recorded during the development of colitis; meanwhile, mice feces were collected every day to examine for soft or loose stools and obvious or occult blood. The Pilamidong test kit (Shanghai Yaji Biotechnology Co., Ltd., Shanghai, China) was used to detect fecal occult blood, according to the manufacturer’s instructions. The mice feces were evaluated according to the standards listed in Table 1, as well as to determine the daily disease activity index (DAI) [21]. After the induction phase, the mice were euthanized. The tissues of the caecum and colon were carefully removed to measure the length of the colon, and then, 1 cm of the distal colon was fixed in a 4% paraformaldehyde solution. The cecum and colon contents and the remainder of the colonic tissue were all immediately stored at −80°C. The mice’s spleen and thymus were carefully removed and weighed. The mass of the mice’s spleen and thymus were divided by their body weight to determine the spleen and thymus index, respectively.

### 2.4. Histopathological Evaluation

Hematoxylin and eosin (H&E) staining was performed to evaluate the colon histopathologic, according to the method of Luzardo-Ocampo et al. [22] with slight modifications. Briefly, the fixed distal colon tissue was first dehydrated using gradient concentrations of alcohol, xylene, and paraffin; then, it was wrapped in paraffin and cut into slices (4 μm) using a rotary microtome (RM2016, Leica Biosystems, Wetzlar, Germany). Finally, the embedded tissue was further dehydrated with alcohol, xylene, and paraffin and then exposed to H&E staining. The histopathological scores were evaluated according to the standards [23] listed in Table 2.

### 2.5. Measurement of Short-Chain Fatty Acids in Cecum Contents

The method to extract short-chain fatty acids (SCFAs) referred to Guo et al. [24] with some modifications. Briefly, 50 mg of the freeze-dried cecum content was added into a 500 μL saturated sodium chloride solution and homogenized after being left at room temperature for 30 min. In total, 20 μL of 10% sulfuric acid was added and fully mixed by vortex for 1 min, followed by being mixed with 800 μL of anhydrous ether and centrifugated at 12,000 r/min, 4 °C for 15 min to extract SCFAs. The supernatant was added into a new centrifuge tube containing 250 mg of anhydrous sodium sulfate to stay for 10 min to remove moisture. Finally, centrifugation was carried out at 12,000 r/min, 4 °C for 15 min to obtain supernatant, which was filtered with a 0.22 μm membrane after being mixed with internal standard (2-ethyl butyric acid) to be tested. The measurement of SCFA content was performed using an Agilent 7890A gas chromatography (GC) system with an HP-INNO WAX column (30 m × 0.32 mm × 0.5 μm) and flame ionization detector (Agilent Technologies Inc., Santa Clara, CA, USA). The GC program was set up according to the description of Li et al. and modified slightly [25], which was expounded as follows: the flow rates of hydrogen, nitrogen, and air were 40 mL/min, 34 mL/min, and 450 mL/min, respectively. The temperature of the injection port and detector were 200 °C and 220 °C, respectively. The split ratio was 10:1. The initial temperature of the column was 90 °C; then, it was raised to 150 °C (3 °C/min) after maintained for 1 min; and then, it was heated up to 200 °C at a speed of 5 °C/min. The quantification of SCFAs was based on external normalized curves of acetic acid, propionic acid, and butyric acid.

### 2.6. Measurement of Cytokine Levels

The contents of tumor necrosis factor-α (TNF-α), interleukin-1β (IL-1β), IL-6, and IL-10 in mice colon tissue were measured using mouse enzyme-linked immunosorbent assay (ELISA) kits (Quanzhou Ruixin Biotechnology Co., Ltd., Quanzhou, China). The mice’s colon tissues were homogenized with precool phosphate-buffered saline (PBS) solution (10 mmol/L, pH 7.0) at a ratio of 1:5 (mg/μL) and then centrifuged at 12,000 r/min for 15 min to obtain supernatant to be measured according to manufacturer’s instructions [26].

### 2.7. Measurement of Myeloperoxidase Activity

The activity of myeloperoxidase (MPO) in mice colon tissue was measured using an ELISA kit (Quanzhou Ruixin Biotechnology Co., Ltd., Quanzhou, China) [26]. The sample preparation was carried out according to the method mentioned in Section 2.6.

### 2.8. Analysis of Gut Microbiota

The mice’s colon contents were used to extract the total genome DNA, in which the 16S rRNA gene was amplified at V3–V4 hypervariable regions using primers 341F (5′-CCTAYGGGRBGCASCAG-3′) and 806R (5′-GGACTACNNGGGTATCTAAT-3′), and polymerase chain reaction (PCR). The PCR program included predegeneration (98 °C for 1 min), cycles (98 °C for 10 s for denaturalization, 50 °C for 30 s for annealing, and 72 °C for 30 s and extended 30 times), and preservation at 72 °C for 5 min. PCR production was sequenced on an Illumina NovaSeq platform. The sequences were analyzed using QIIME (Quantitative Insights Into Microbial Ecology) software (Version 1.7.0), and internal Perl scripts were used to investigate α and β diversity. The analysis of gut microbiota referred to Li et al. [27].

### 2.9. Statistical Analysis

The results are presented as mean ± standard deviation. The data were analyzed via one-way analysis of variance (ANOVA), followed by the Duncan test. Differences were considered statistically significant at *p* < 0.05.

## 3. Results

### 3.1. L. plantarum, COS, and Synbiotics Alleviated the DSS-Induced Colitis Symptoms

A total of 366 colonies with typical lactic acid bacteria morphology were observed on the MRS plate, in which the Gram-positive and catalase-negative isolates were identified via molecular identification. A strain of *Lactiplantibacillus plantarum* was obtained as an endogenous *L. plantarum* from the mice. The anti-inflammatory activity of endogenous and exogenous *L. plantarum*, and combined synbiotics of *L. plantarum* with COS were investigated using DSS-induced acute colitis mice. As shown in Figure 1b, the mice lost weight from the third day after DSS treatment, and the weight dropped sharply on the last day of induction, with decreasing percentages of 13.93% ± 3.37%, 4.52% ± 1.94%, 4.39% ± 2.65%, 4.45% ± 3.64%, 3.64% ± 2.32%, and 3.16% ± 1.33% in the MOL, LP-M, LP-P, COS, MC, and PC groups compared with the NC group, respectively. The results suggested that *L. plantarum*, COS, and the synbiotics significantly inhibit (*p* < 0.05) the DSS-induced weight loss in mice, while there was no significant difference among the five experimental groups.

The disease activity index (DAI) is a composite score relating to the changes in weight and stool during colitis development and is used to assess the clinical symptoms of colitis. The continuously rising DAI illustrated in Figure 1c was associated with body weight loss, diarrhea, and bloody stools in mice. The mice in the MOL group began to have loose stools on the second day after DSS induction, which was not observed in other experimental groups. As the induction of DSS continued, diarrhea and blood in the stools of the MOL mice were progressively more severe and the DAI score increased. LP-M, LP-P, COS, and the synbiotics all could alleviate diarrhea and bleeding of colitis mice, resulting in significantly lower DAI scores than the MOL group from the second day. There was no significant difference in the DAI scores between the LP-M and MC groups, while the DAI score in the PC group was significantly lower than that in the LP-P group in the last three days of induction (*p* < 0.05), indicating that COS had a better enhancing effect on colitis relief by exogenous *L. plantarum*, compared with endogenous *L. plantarum*.

Figure 1d showed the representative macroscopic photograph of the colon tissue, and their length was summarized in Figure 1e. DSS caused the shortening of colon length, swelling, and bleeding of the colon wall, with unshaped content in the colon, and these injuries were mitigated by *L. plantarum*, COS, and synbiotic treatment. The mice’s colon lengths decreased significantly from 9.08 ± 0.42 cm in the NC group to 5.59 ± 0.39 cm in the MOL group (*p* < 0.05). Compared with the MOL group, the improvement effects of *L. plantarum*, COS, and the synbiotics on colon length were all significant(*p* < 0.05), which were 7.47 ± 0.88 cm, 6.87 ± 1.40 cm, 6.82 ± 1.42 cm, 6.89 ± 0.45 cm, and 7.47 ± 0.82 cm in the LP-M, LP-P, COS, MC, and PC group, respectively, while the data in these groups showed no statistically significant difference.

In addition, DSS affected the mice organs, among which the spleen and thymus were the most negatively impacted organs. The results in Figure 1f,g indicated that the spleen index of MOL group mice greatly rose when compared with the NC group, while the thymus index dramatically decreased, which was connected to spleen enlargement and thymus atrophy, reveling the immune abnormalities in mice with colitis. *L. plantarum*, COS, and the synbiotics inhibited the changes in the spleen and thymus of mice. The spleen index of the LP-M, COS, MC, and PC group mice did not change significantly from that of the NC group mice, demonstrating that endogenous *L. plantarum* was not influenced by COS but that the immunomodulation effect of exogenous *L. plantarum* was improved by combining with COS to form synbiotic. However, for the thymus index, there was not a noticeable distinction in the results of *L. plantarum*, COS, and the synbiotics.

### 3.2. L. plantarum, COS, and Synbiotics Alleviated the Colon Damage in DSS-Induced Colitis Mice

**Figure 2:**
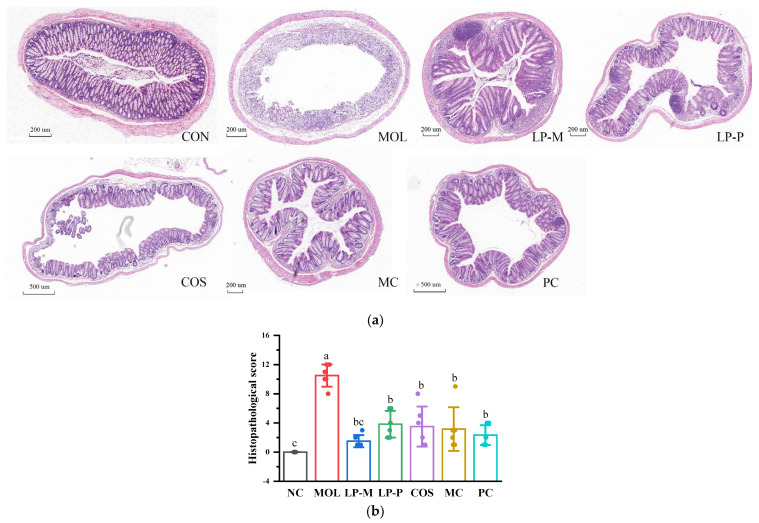
*L. plantarum*, COS, and synbiotics alleviated colon damage in DSS−induced colitis mice. (**a**) Representative hematoxylin and eosin (H&E) staining images of distal colon tissues. (**b**) The histopathological score based on H&E staining (n = 6). Statistical analysis was performed via one−way ANOVA with Duncan Test. The different letters above the columns indicated a significant difference (*p* < 0.05).

DSS severely disrupted the colon barrier in mice, manifested as epithelial cell destruction, crypt loss, and inflammatory cell infiltration (Figure 2a). Compared with NC group, the histopathological score of MOL group increased significantly (*p* < 0.05). Both *L. plantarum*, COS, and the synbiotics recovered mucosal damage, and endogenous *L. plantarum* restored the histopathological score to a level that was insignificantly different from normal mice. LP-P, COS, and the synbiotics also remarkably inhibited the damage of DSS to the colon (*p* < 0.05), while there were no notable distinctions in histopathological scores between the NC, LP-P, COS, MC, and PC groups (Figure 2b).

### 3.3. L. plantarum, COS, and Synbiotics Improved the Short-Chain Fatty Acids in Cecum Contents of DSS-Induced Colitis Mice

**Figure 3:**
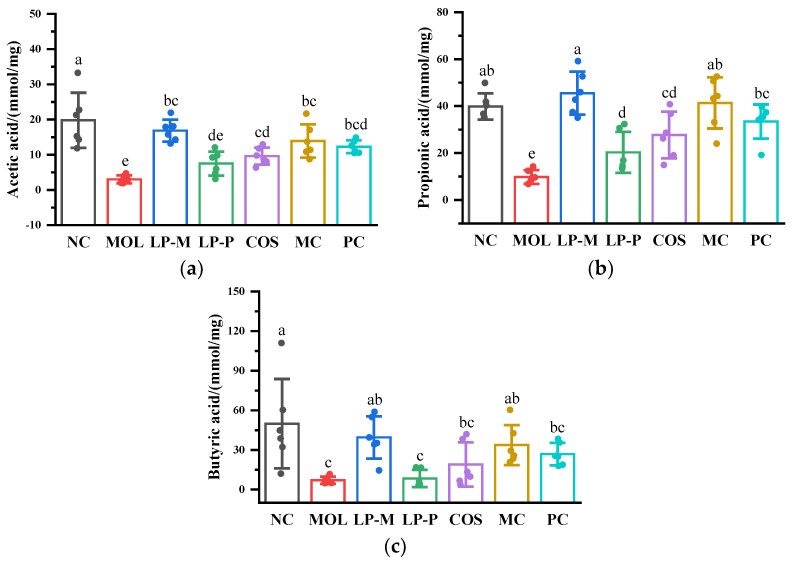
*L. plantarum*, COS, and synbiotics improved the short−chain fatty acids in the cecum contents of DSS−induced colitis mice (n = 6). (**a**) Acetic acid. (**b**) Propionic acid. (**c**) Butyric acid. Statistical analysis was performed via one−way ANOVA with Duncan Test. The different letters above the columns indicated a significant difference (*p* < 0.05).

As shown in Figure 3, the levels of acetic acid, propionic acid, and butyric acid were significantly lower in the MOL group than that in the NC group (*p* < 0.05), and *L. plantarum*, COS, and the synbiotics all contributed to the increase in SCFA content. Endogenous *L. plantarum* LP-M and synbiotics combined with COS showed superior improvement in SCFAs, while there was no statistically significant difference between the LP-M and MC groups. Meanwhile, LP-M up-regulated the acetic acid content to a level that was not notably different from that in the NC group; however, a significant difference was observed between the MC and NC groups, indicating that endogenous *L. plantarum* more effectively promoted acetic acid than the endogenous symbiotics. Differently, the synbiotics consisted of exogenous *L. plantarum* LP-P, and COS had an additional ability to elevate SCFAs. The mean concentrations of acetic acid, propionic acid, and butyric acid in the cecum contents of the mice in the PC group were, respectively, 38.82%, 39.31%, and 68.73% higher than those in the LP-P group, and 21.49%, 17.11%, and 29.48% higher than those in the COS group, demonstrating that the synbiotic promoted SCFAs more effectively than *L. plantarum* and COS alone.

### 3.4. L. plantarum, COS, and Synbiotics Influenced Cytokine Levels Positively in the Colon of DSS-Induced Colitis Mice

**Figure 4:**
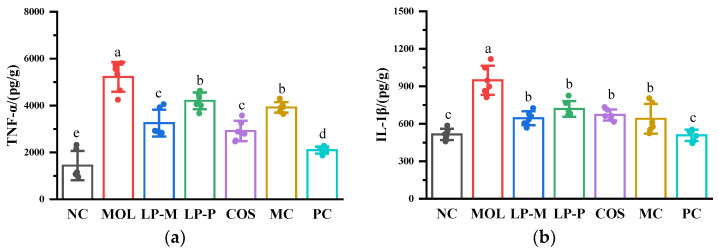

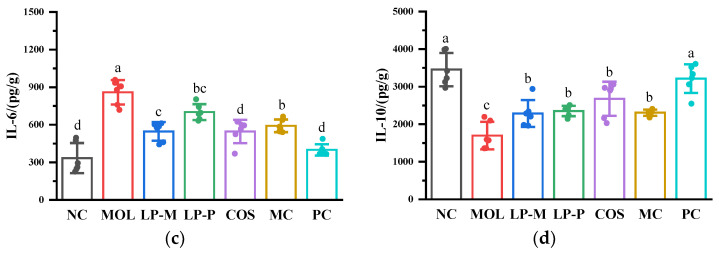
*L. plantarum*, COS, and synbiotics influenced cytokine levels positively in the colon of DSS−induced colitis mice (n = 6). (**a**) TNF−α. (**b**) IL−1β. (**c**) IL−6. (**d**) IL−10. Statistical analysis was performed via one−way ANOVA with Duncan Test. The different letters above the columns indicated a significant difference (*p* < 0.05).

The cytokine levels in the mouse colons were negatively impacted by DSS, which was proved by significantly (*p* < 0.05) higher pro-inflammatory cytokine TNF-α, IL-1β, and IL-6 levels and notably (*p* < 0.05) lower anti-inflammatory cytokine IL-10 levels in the mice of the MOL group than in the NC group (Figure 4). *L. plantarum*, COS, and the synbiotics positively influenced the cytokine levels, resulting in a noticeable (*p* < 0.05) inhibition of the changes in TNF-α, IL-1β, IL-6, and IL-10. The levels of TNF-α and IL-6 in the MC group were higher than that in the LP-M group, suggesting that a compound of COS and synbiotics was disadvantageous to the anti-inflammatory properties of endogenous *L. plantarum* LP-M. However, pro-inflammatory factors were at remarkably (*p* < 0.05) lower levels in the PC group than in the LP-P and COS groups, while the IL-10 level was outstandingly (*p* < 0.05) higher in the PC group than in the LP-P and COS groups. The results indicated that combining LP-P and COS to formulate synbiotics could function as a potential approach for increasing the anti-inflammatory capacity of exogenous *L. plantarum* and COS.

### 3.5. L. plantarum, COS, and Synbiotics Inhibited the MPO Activity in the Colon of DSS-Induced Colitis Mice

**Figure 5:**
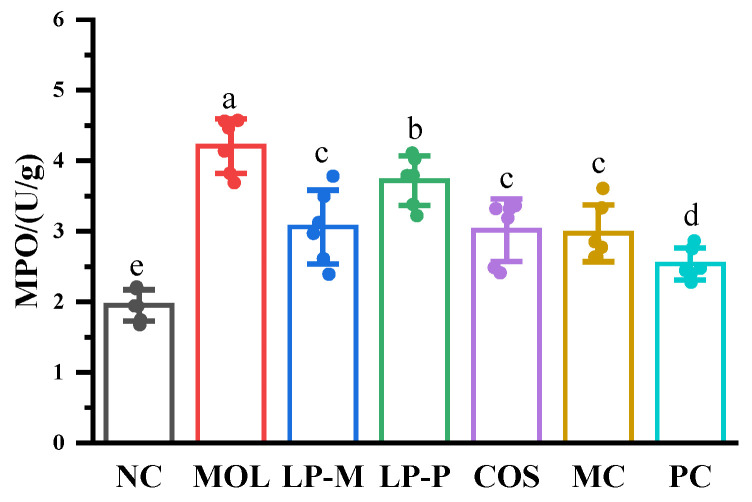
*L. plantarum*, COS, and synbiotics inhibited the MPO activity in the colon of DSS−induced colitis mice (n = 6). Statistical analysis was performed via one−way ANOVA with Duncan Test. The different letters above the columns indicated a significant difference (*p* < 0.05).

The result in Figure 5 demonstrated the increasing MPO activity in mice of MOL group, compared with that in NC group, which was suppressed by *L. plantarum*, COS, and the synbiotics. Although there was no statistically difference in MPO activity among the LP-M, COS, and MC groups, it was lower in the PC group compared with the LP-P and COS groups, indicating that LP-P and COS had synergistic effects in inhibiting MPO alterations.

### 3.6. L. plantarum, COS, and Synbiotics Restored the Gut Microbiota in Colon Contents of DSS-Induced Colitis Mice

**Figure 6:**
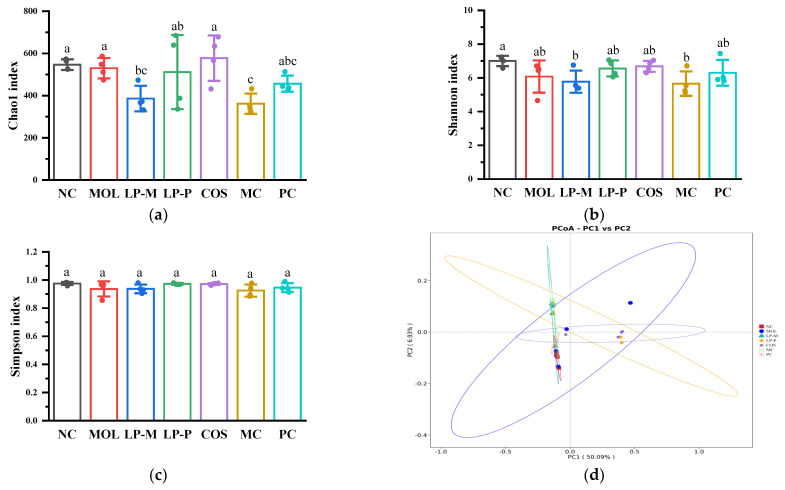

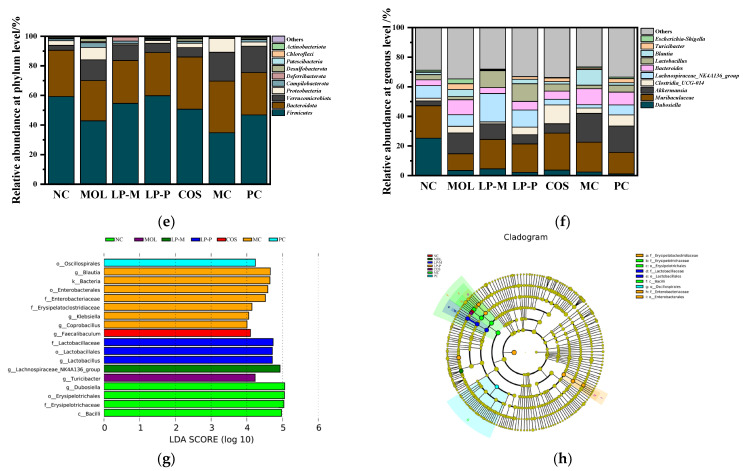
*L. plantarum*, COS, and synbiotics restored the gut microbiota in colon contents of DSS−induced colitis mice (n = 4). (**a**–**c**) The α−diversity of gut microbiota illustrated by the Chao1 index, Shannon index, and Simpson index, respectively. Statistical analysis was performed via one−way ANOVA with Duncan Test. The different letters above the columns indicate a significant difference (*p* < 0.05). (**d**) β−diversity assessed via PCoA analysis based on weighted UniFrac distances. (**e**) Relative abundance of the microbiota of mouse colon contents at the phylum level. (**f**) Relative abundance of the microbiota of mouse colon contents at the genus level. (**g**) The linear discriminant analysis (LDA, threshold 4). (**h**) Generated classification map.

Figure 6a–c displayed the mice α-diversity of gut microbiota. The Chao1, Shannon, and Simpson indexes in the MOL group were not significantly different from those in the NC group. Meanwhile, the structure of the gut bacterial communities was investigated using a Principal Co-ordinates Analysis (PCoA) based on weighted UniFrac distances to assess the β-diversity among treatment groups. As shown in Figure 6d, the samples from DSS-induced colitis mice moved towards the PC1 positive axis and the PC2 positive axis, with an obvious dispersion in the MOL group, which was inhibited by LP-M, LP-P, COS, and synbiotic interventions. It was worth noting that the samples in the PC group were closer to the NC group compared with the LP-P and COS groups, which minimized the tendency of the samples to move toward the PC1- and PC2-positive axes, indicating that the COS and LP-P had synergistic effects in restoring the structure of the gut bacterial communities in mice with colitis.

The dominant bacteria in the mice gut at the phylum level were *Firmicutes* and *Bacteroidota*, with a relative abundance sum of 90.55% ± 0.97% in NC group mice, while it dropped down to 70.04% ± 19.94% in the MOL group (Figure 6e). *Verrucomicrobiota* saw the greatest rise in relative abundance in the MOL group compared with normal mice, with an increase of 76.72%, followed by *Desulfobacterota*, whose relative abundance increased by 67.13%. *Proteobacteria*, *Campilobacterota*, *Deferribacterota*, and *Patescibacteria*’s relative abundances also increased, indicating that DSS also had a facilitative impact on the six other non-major bacteria in the mouse gut at the phylum level. Overall, phylum-level changes in the gut microbiota were suppressed by *L. plantarum*, COS, and the synbiotics. However, the relative abundance of *Verrucomicrobiota* was larger in the MC and PC groups than in the MOL group. At the genus level (Figure 6f), the abundant genera in the mouse gut microbiota were *Dubosiella* and *Muribaculaceae*, but in the MOL group, *Dubosiella* and *Muribaculaceae* decreased by 86.42% and 48.38%, respectively, while *Akkermansia* became the dominant genera with relative abundance increased by 76.71%. In addition, an increase in *Clostridia_UCG-014*, *Bacteroides*, *Blautia*, *Turicibacter*, and *Escherichia-Shigella* and a decrease in *Lachnospiraceae_NK4A136_group* and *Lactobacillus* were observed in the MOL group. *L. plantarum*, COS, and the synbiotics all showed the ability to inhibit the changes of *Muribaculaceae*, *Lactobacillus*, *Turicibacter*, and *Escherichia-Shigella*. Similar to the result, *Akkermansia*, belonging to the phylum *Verrucomicrobiota*, was higher in the synbiotics intervention mice than in the MOL group.

Figure 6g,h show the linear discriminant analysis (LDA) and generated classification map, respectively, used to perform the LEfSe to investigate the biomarker, with statistical differences among the different groups from the phylum to the genus levels in the gut microbiota. The genera of the biomarkers in the NC, MOL, LP-M, LP-P, COS, and PC groups were *Dubosiella*, *Turicibacter*, *Lachnospiraceae_NK4A136_group*, *Lactobacillus*, *Faecalibaculum*, and *Oscillospirales*, respectively. *Blautia*, *Klebsiella*, and *Coprobacillus* were the biomarkers in the MC group.

### 3.7. The Differential Analysis of the Effects of COS on Gut Microbiota Regulation in DSS-Induced Colitis Mice of Endogenous and Exogenous L. plantarum

**Figure 7:**
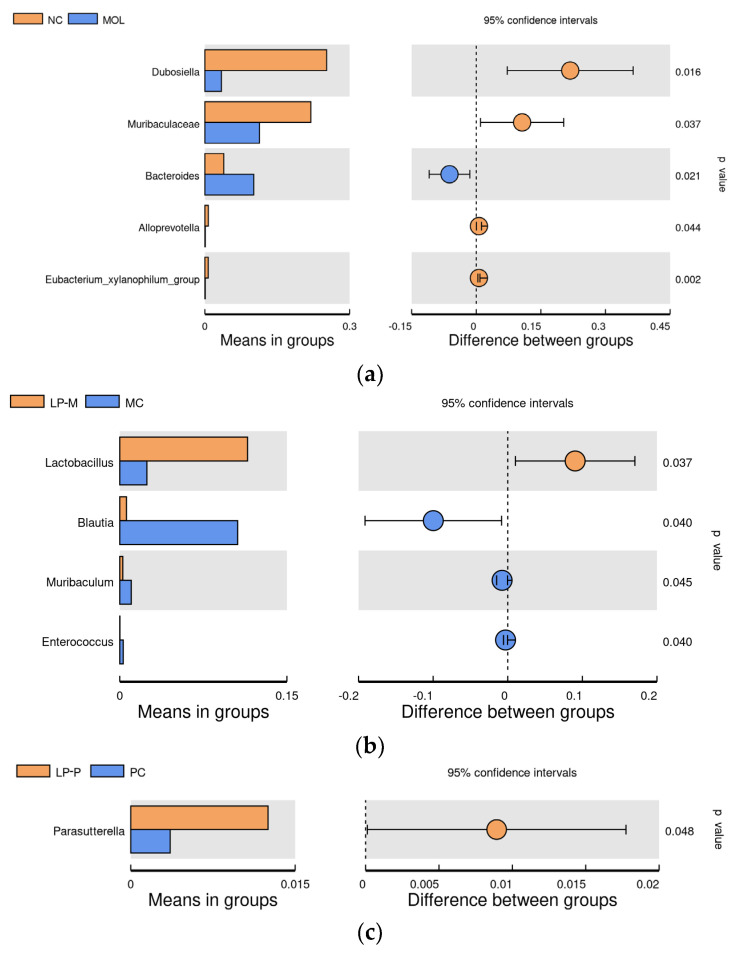
The *T*−test analysis−identified gut microbiota between NC and MOL groups at genus level (**a**). The differential analysis of the effects of COS on the gut microbiota regulation in DSS−induced colitis mice of endogenous (**b**) and exogenous (**c**) *L. plantarum* using *T*−test analysis (n = 4).

The results in Figure 7a showed that the relative abundance of *Dubosella*, *Muribaculaceae*, *Alloprevotella*, and *Eubacterium_xylanophilum_group* in mice of the MOL group were significantly (*p* < 0.05) lower than that in NC group, while the *Bacteroides* remarkably increased (*p* < 0.05) after DSS induction. Meanwhile, *T*-test (*p* < 0.05) was utilized to analyze differences of taxonomic differences in the gut microbiota at the genus level between the LP-M and MC groups and between the LP-P and PC groups to explore the effect of COS on the microbiota regulation of endogenous and exogenous *L. plantarum*. As shown in Figure 7, the relative abundance of *Lactobacillus* decreased significantly in the MC group, compared with the LP-M group, while the *Blautia*, *Muribaculum*, and *Enterococcus* showed a notable increases. However, compared with LP-P administration, only a reduction in *Parasutterella* was detected after being gavaged by the synbiotic combination of exogenous *L. plantarum* and COS.

## 4. Discussion

The limited effect and adverse reactions such as immunological tolerance and loss of drug resistance are all problems with traditional therapies for inflammatory bowel disease (IBD) [28]. As a result, a lot of research has focused on microecological treatments, such as probiotics, prebiotics, and the synbiotics, as supplemental or alternative medications for the treatment of IBD. In this work, synbiotics were formulated using COS as a prebiotic, endogenous *L. plantarum* isolated from mice feces, and exogenous *L. plantarum* isolated from pickles, as probiotics. The effects of *L. plantarum*, COS, and the synbiotics on DSS-induced acute colitis mice were investigated, as well as the influence of COS on the activities of endogenous and exogenous *L. plantarum*. Both endogenous and exogenous *L. plantarum*, COS, and the synbiotics demonstrated the ability to alleviate colitis symptoms, including preventing weight loss, and loose and bloody stools; hindering splenomegaly and thymic atrophy; restoring colon status and length; and lowering colon histopathological scores. Overall, the effects of endogenous *L. plantarum* and its synbiotic were not significantly different, whereas the exogenous *L. plantarum* synbiotic presented more notable effects on mice colitis than single *L. plantarum*. Furthermore, the impacts of *L. plantarum*, COS, and the synbiotics were researched based on intestinal metabolism, immunity, and microbiota. Additionally, the reasons for the different consequences in COS on endogenous and exogenous *L. plantarum* were analyzed.

*L. plantarum*, COS, and the synbiotics enhanced the short-chain fatty acid (SCFA) levels in the cecum contents of colitis mice. There was no discernible difference between endogenous *L. plantarum* LP-M and the synbiotic combination of LP-M and COS in improvement to SCFAs. However, the effect of synbiotic consisting of exogenous *L. plantarum* LP-P and COS on SCFAs was superior to exogenous *L. plantarum* LP-P. Particularly, the propionic acid level in the PC group was higher than that in the LP-P group (*p* < 0.05). SCFAs are the critical intestinal metabolite and essential parameter for assessing the effect of microecological agents on IBD. Acetic acid, propionic acid, and butyric acid all play roles in maintaining the integrity of intestinal barrier [29,30,31], and butyric acid is a vital nutrient for colonic epithelial cells [32]. Moreover, SCFAs further assist with proper immune system function [33], maintain the balance of the gut microbiota, strengthen gastrointestinal motility, and contribute to digestion and absorption [34]. The findings of the present study revealed that *L. plantarum*, COS, and the synbiotics all significantly increased the levels of acetic acid, propionic acid, and butyric acid, indicating that they possibly contributed to maintaining the balance of the intestinal barrier by influencing intestinal metabolism, therefore mitigating the damage to the intestinal barrier caused by DSS and relieving colitis symptoms.

Abnormal immune responses in the gut are a feature of colitis [35], which is characterized by the increase of inflammatory cytokines (TNF-α, IL-1β, IL-6, etc) and decrease of anti-inflammatory cytokines, such as IL-10, and these changes are observed in patients with IBD [36] and DSS-induced colitis mice [37]. The results of the current study revealed the changes in cytokine levels resulting from immunological abnormalities caused by DSS in the gut were attenuated by *L. plantarum*, COS, and the synbiotics. It was attainable that the synergistic effect of COS and exogenous *L. plantarum* was attributed to elevating the anti-inflammatory capacity of exogenous *L. plantarum* because the synbiotic combined with LP-P and COS, with greater improvement to cytokines levels than LP-P, was the most effective modulator of cytokines of all the samples tested. The synergistic effect of COS and LP-P was also supported by the results of MPO activity, which was much lower in the PC group than in the LP-P group. Epithelial damage caused by DSS directly activates the recruitment of neutrophils at the site of inflammation, producing MPO, a key enzyme associated with the catalytic synthesis of strong cytotoxic oxidants, and thus, MPO activity is seen as a reflection of neutrophil infiltration during inflammation and correlates linearly with neutrophil infiltration [38].

Numerous intestinal microorganisms are found in the host gut. Gut microbiota produces short-chain fatty acids, polysaccharides, bacteriocins, vitamin K, and amino acids through the functional transformation of diet [39]. In addition, gut microbiota prevents the physical barrier of the intestinal tract from harmful bacteria and metabolites to strengthen the barrier’s protective ability and play roles in the activation of regulatory T cells and anti-inflammatory cytokines to maintain immune homeostasis [39,40]. Patients with IBD frequently experience dysbiosis of the gut microbiota, which manifests as an increase in pathogenic bacteria and a reduction in beneficial bacteria [41]. The capacity to restore gut microbiota is an essential indicator for assessing the effect of microecological agents on IBD. In the present study, there was no significant change in the α-diversity of the gut microbiota in the DSS-induced colitis mice, but the β-diversity evidenced that the fundamental composition of the gut microbiota was dissimilar in normal and colitis mice. *L. plantarum*, COS, and the synbiotics all exhibited the ability to restore the gut microbiota, mainly converging in the recovery of the basic composition of the gut microbiota and the major genera in normal mice.

It was observed that the relative abundance of beneficial bacteria *Dubosiella*, *Muribaculaceae*, *Lachnospiraceae_NK4A136_group*, and *Lactobacillus* was reduced by DSS and that the abundance of pathogenic genera *Clostridia UCG-014*, *Bacteroides*, *Escherichia-Shigella*, and *Turicibacter* was increased. *Dubosiella* has been proposed to be a potentially beneficial bacteria that can alleviate colitis in mice [42]. The abundance of *Muribaculaceae* is strongly correlated with propionic acid concentration [43] and negatively correlated with pro-inflammatory cytokines TNF-α, IL-6, and IL-1β [44]. The *Lachnospiraceae_NK4A136_group* reduces intestinal inflammation and diarrhea [45] and facilitates the recovery of colitis [46]. *Lactobacillus* is involved in regulating intestinal inflammation and the epithelial barrier of the host by affecting tryptophan metabolism [47]. *Clostridia UCG-014* is shown to be adversely linked with acetic acid and butyric acid [48]. *Bacteroides*, a potentially pathogenic bacterium, has unfavorable effects on the mucus layer [49] and is associated with the development of colitis [50]. *Escherichia-Shigella* is a pathogenic bacterium that is contributed to the severity of colitis [51]. *Turicibacter is* positively correlated with pro-inflammatory cytokines [52], and enrichment of *Turicibacter* has a negative effect on the Shannon index of gut microbiota [53]. *L. plantarum*, COS, and the synbiotics inhibited the decrease in *Muribaculaceae* and *Lactobacillus* and showed a poor effect on the rise of *Dubosiella*, while *Lachnospiraceae_NK4A136_group* was up-regulated by *L. plantarum*. Meanwhile, the relative abundance of *Turicibacter* and *Escherichia-Shigella* were also inhibited by *L. plantarum*, COS, and the synbiotics.

The LefSe analysis indicated that *Dubosiella*, *Turicibacter*, *Lachnospiraceae_NK4A136_group*, *Lactobacillus*, *Faecalibaculum*, and *Oscillospirales* were notably abundant in mice in the NC, MOL, LP-M, LP-P, COS, and PC groups, respectively. *Blautia*, *Klebsiella*, and *Coprobacillus* were the biomarkers in the MC group. *Faecalibaculum* is positively correlated with fecal levels of acetic acid, propionic acid, and total acids [54] and negatively correlated with inflammatory cytokines [55]. *Oscillospirales* is a potential candidate for the next generation of probiotics that encourages the formation of SCFAs [56]. *Coprobacillus* is thought to maintain intestinal stability and to resist colonization by *Clostridium difficile* [57]. *Klebsiella* can promote colitis [58] and stimulate T cell differentiation and inflammatory cytokines [59]. However, it is still unclear if *Blautia* is effective for treating colitis because it helps to enhance the creation of SCFAs [60], while is concentrated in UC patients’ gut [61] and positively correlates with IL-1β, IL-6, and TNF-α [62]. The results presented in Section 3.6. suggested that the effect of endogenous and exogenous *L. plantarum* on the gut microbiota of mice was altered after combination with COS. For exogenous *L. plantarum* LP-P, there was an alteration from one beneficial genus to another, but for endogenous *L. plantarum* LP-M, there was a change from one helpful genus to one beneficial, one potentially harmful, and one controversial strain. The anti-inflammatory impact of endogenous *L. plantarum* in conjunction with COS possibly had been diminished as a result of this variation.

*Parasutterella* was the only significant genera detected when the mice gut microbiota was compared between a synbiotic combination of COS and exogenous *L. plantarum*, and single *L. plantarum* intervention. *Parasutterella* is negatively correlated with propionic acid levels in mice [63] and positively correlated with the pro-inflammatory cytokines [64], suggesting that *Parasutterella* promotes the development of colitis by stimulating inflammatory cytokines. The effects of exogenous synbiotics on lowering the relative abundance of *Parasutterella* could be part of the explanation for the decreased concentrations of TNF-α, IL-1β, and IL-6 observed in the colon of mice in the PC group. Due to the low relative abundance, further investigation is necessary to determine if *Parasutterella* possessed an essential function in the anti-inflammatory capacity, although it might be a research focus to examine the impact of COS on exogenous *Lactiplantibacillus plantarum* LP-P.

## 5. Conclusions

This study demonstrated that the *L. plantarum* strain isolated from mice feces and a pickles, and their synbiotics combined with COS all showed anti-inflammatory activity. However, the anti-inflammatory ability of exogenous synbiotics was superior to that of exogenous *L. plantarum*; the same conclusion was not drawn in the comparison between endogenous synbiotics and endogenous *L. plantarum*. The exogenous synbiotics inhibited the MPO activity and reconstructed the composition of gut microbiota more effectively. Therefore, combination with COS to form a synbiotic was an effective way to enhance the anti-inflammatory activity of exogenous *L. plantarum* LP-P.

## Figures and Tables

**Table 1:** The evaluation standards of disease activity index.

Score	Weight Loss	Stool Consistency	Bleeding
0	<1%	Normal	Normal
1	1–5%	Slightly soft	Occult blood and weak positive
2	5–10%	Soft	Occult blood and positive
3	10–20%	Loose	Visible blood
4	>20%	Diarrhea	Gross bleeding

**Table 2:** The standards of histopathological evaluation.

Score	Epithelial Cell Destruction	Crypt Loss	Inflammatory Cell Infiltration
0	Normal	Normal	Normal
1	Localized and mild	Localized and mild	Localized and mild
2	Localized and moderate	Localized and moderate	Localized and moderate
3	Extensive and moderate	Extensive and moderate	Extensive and moderate
4	Extensive and severe	Extensive and severe	Extensive and severe

## Data Availability

The data are contained within the article.
